# Review of Pharmacokinetics and Pharmacogenetics in Atypical Long-Acting Injectable Antipsychotics

**DOI:** 10.3390/pharmaceutics13070935

**Published:** 2021-06-23

**Authors:** Francisco José Toja-Camba, Nerea Gesto-Antelo, Olalla Maroñas, Eduardo Echarri Arrieta, Irene Zarra-Ferro, Miguel González-Barcia, Enrique Bandín-Vilar, Victor Mangas Sanjuan, Fernando Facal, Manuel Arrojo Romero, Angel Carracedo, Cristina Mondelo-García, Anxo Fernández-Ferreiro

**Affiliations:** 1 Pharmacy Department, University Clinical Hospital of Ourense (SERGAS), Ramón Puga 52, 32005 Ourense, Spain; kikotoja@gmail.com; 2 Clinical Pharmacology Group, Institute of Health Research (IDIS), Travesía da Choupana s/n, 15706 Santiago de Compostela, Spain; irene.zarra.ferro@sergas.es (I.Z.-F.); miguel.gonzalez.barcia@sergas.es (M.G.-B.); enriquebandinvilar@gmail.com (E.B.-V.); victor.mangas@uv.es (V.M.S.); 3 Genomic Medicine Group, CIMUS, University of Santiago de Compostela, 15782 Santiago de Compostela, Spain; nerea.gesto@rai.usc.es (N.G.-A.); olalla.maronas@hotmail.com (O.M.); 4 Pharmacy Department, University Clinical Hospital of Santiago de Compostela (SERGAS), 15706 Santiago de Compostela, Spain; eduardo.echarri.arrieta@sergas.es; 5 Department of Pharmacy and Pharmaceutical Technology and Parasitology, University of Valencia, 46100 Valencia, Spain; 6 Interuniversity Research Institute for Molecular Recognition and Technological Development, Polytechnic University of Valencia-University of Valencia, 46100 Valencia, Spain; 7 Psychiatry Department, University Clinical Hospital of Santiago de Compostela (SERGAS), Travesía da Choupana s/n, 15706 Santiago de Compostela, Spain; ferfacal92@hotmail.com (F.F.); Manuel.Arrojo.Romero@sergas.es (M.A.R.); 8 Institute of Health Research (IDIS), Travesía da Choupana s/n, 15706 Santiago de Compostela, Spain; 9 Genomic Medicine Group, Biomedical Research Center of Rare Diseases (CIBERER), University of Santiago de Compostela, 15782 Santiago de Compostela, Spain; 10 Galician Foundation of Genomic Medicine, Foundation of Health Research Institute of Santiago de Compostela (FIDIS), SERGAS, 15706 Santiago de Compostela, Spain

**Keywords:** population pharmacokinetic models, pharmacogenetics, LAI, risperidone, paliperidone, aripiprazole, CYP2D6, antipsychotics

## Abstract

Over the last two decades, pharmacogenetics and pharmacokinetics have been increasingly used in clinical practice in Psychiatry due to the high variability regarding response and side effects of antipsychotic drugs. Specifically, long-acting injectable (LAI) antipsychotics have different pharmacokinetic profile than oral formulations due to their sustained release characteristics. In addition, most of these drugs are metabolized by *CYP2D6*, whose interindividual genetic variability results in different metabolizer status and, consequently, into different plasma concentrations of the drugs. In this context, there is consistent evidence which supports the use of therapeutic drug monitoring (TDM) along with pharmacogenetic tests to improve safety and efficacy of antipsychotic pharmacotherapy. This comprehensive review aims to compile all the available pharmacokinetic and pharmacogenetic data regarding the three major LAI atypical antipsychotics: risperidone, paliperidone and aripiprazole. On the one hand, CYP2D6 metabolizer status influences the pharmacokinetics of LAI aripiprazole, but this relation remains a matter of debate for LAI risperidone and LAI paliperidone. On the other hand, developed population pharmacokinetic (popPK) models showed the influence of body weight or administration site on the pharmacokinetics of these LAI antipsychotics. The combination of pharmacogenetics and pharmacokinetics (including popPK models) leads to a personalized antipsychotic therapy. In this sense, the optimization of these treatments improves the benefit–risk balance and, consequently, patients’ quality of life.

## 1. Introduction

Schizophrenia is a severe mental disorder that is presented clinically heterogeneously in patients. It is estimated that approximately 0.7–1% of the world population suffer from this condition, currently affecting 24–25 million people worldwide according to data from the World Health Organization [1,2,3]. Mainly, it causes disorder of behavior, thoughts, perception and emotions. The core features are positive symptoms (delusions, hallucinations and disorganized speech), negative symptoms (such as abulia, anhedonia and social withdrawal), cognitive impairment (mainly in speed of mental processing, working memory and executive functions) and affective symptoms (i.e., anxiety and depression) [4]. There are well-defined instruments such as the Brief Psychiatric Rating Scale (BPRS), the Scale for the Assessment of Positive Symptoms (SAPS), the Scale for the Assessment of Negative Symptoms (SANS) or the Positive and Negative Symptoms Scale (PANSS) to measure these positive and negative symptoms as well as general psychopathology. In clinical studies, reductions in these scales have been used to define treatment response [5]. There are several theories that try to explain the pathophysiology of this complex and highly polygenic disease, including brain structure defects or neurochemical alterations [6]. Recently Genome Wide Association Studies have supported some of those classic neurochemical hypotheses such as the dopaminergic and glutamatergic from a genetic perspective [7]. Nowadays, the pharmacological treatment of schizophrenia is based on antipsychotics, a family of drugs which are classified into two separate groups: first generation or typical (i.e., haloperidol, chlorpromazine) and second generation or atypical (i.e., risperidone, paliperidone, aripiprazole). Both of them present affinity toward D2 receptors and reduce positive symptoms. Atypical antipsychotics form a heterogenous group of drugs with regard to their different receptor affinity. In addition to D2 receptor affinity, they have other pharmacological actions involving different signaling pathways such as serotoninergic receptors (5-HT_2A_,5-HT_1A_ and 5-HT_2C_) that could be the main cause of their safer profile compared to typical antipsychotics [8].

The effectivity of these drugs is far from expected. Treatment resistance requires dose optimization that is based on a trial-and-error method caused by the current inability to predict response to treatment; this added to severe and frequent adverse events are commonly the reasons that lead the patients to discontinue antipsychotic therapies. The main handicap of oral antipsychotics formulations is adherence. Many factors have influence on non-adherence such as forgetting doses of medication, adverse events during therapy and poor insight that condition drug intake. According to various studies, which define adherence as taking medication at least 75% of the time, non-adherence is around 50% of treated patients [9,10]. This problem has been partially solved since typical long-acting injectable (LAI) antipsychotics were released. Later, arrival of atypical LAI antipsychotics introduced an improvement in the safety profile of these drugs, which are administered at long intervals of time (from 2 weeks to 3 months). At this point, the tandem of pharmacogenetics and pharmacokinetics plays a key role, and personalized prescription of antipsychotics improves the safety and efficacy of pharmacotherapy. Combining pharmacogenetics with therapeutic drug monitoring (TDM) of LAI antipsychotics would be a great tool to predict and anticipate patient response in order to achieve mental wellness and minimize side effects. Furthermore, population pharmacokinetic (popPK) models should be considered as a useful tool to understand the relationship between patient characteristics and drug exposure.

Among all over the genes involved in pharmacokinetics of drugs, the *Cytocrome P450* family is, by far, the most important in terms of interindividual variability. Most of drugs that are commonly used in psychiatry are *CYP2D6*-dependent [11,12], an enzyme encoded from a highly polymorphic gene. Nowadays, there are already 145 alleles described for CYP2D6. These alleles can have different functions, frequency and clinical impact on metabolism across different populations [13,14,15]. Depending on allele combination, it is possible to differentiate the following metabolizer status [16,17,18,19,20,21]: extensive or normal metabolizer (EM/NM): their metabolic pathway is not altered; poor metabolizers (PM): the activity enzyme is significantly reduced or absent; intermediate metabolizer (IM): those who can metabolize substances in lower ratios compared to an EM but better than a PM. Rapid or ultrarrapid metabolizer (UM): enzymatic hyperactivity compared to EM. Those attracting more interest are PM, IM and UM due to their risk of developing adverse reactions or lack of efficacy in clinical practice [22,23,24], so currently development in this area of knowledge holds great promise for reducing time between diagnosis and seeking an effective treatment. Lastly, a mismatch has been observed between the genotype-based prediction of *CYP450*-mediated drug metabolism and the true capacity of an individual to metabolize drugs (phenotype) due to non-genetic factors, which is called phenoconversion [25]. 

Compared to classical pharmacokinetics, where several samples at different time points are collected from a patient to estimate their individual pharmacokinetic parameters (clearance, distribution volume, half-life, etc.), population pharmacokinetics (popPK) analyzes the pooled data available of several patients in order to estimate pharmacokinetic parameter values and their variance, identify covariates and study the random effects (parameter variance which cannot be explained by covariates). All this information implemented in a mathematical model (popPK models) plus Bayesian computational methods allows us to estimate our patients’ individual pK parameters, which can be a very helpful tool to individualize dosing and optimize the treatment efficacy and safety. 

The three major prescribed LAI-antipsychotics are risperidone, paliperidone and aripiprazole, while LAI-olanzapine prescription has been decreasing over time [26]. Our aim is to comprehensively review all the available literature about these three major prescribed LAI-antipsychotics pharmacogenetics and pharmacokinetics, including the existing popPK models as a crucial tool for predicting drug exposure, focusing on risperidone, paliperidone and aripiprazole.

## 2. Long-Acting Risperidone

Risperidone is an atypical antipsychotic derived from benzisoxazole with potent D2 and 5-HT2 receptor antagonism. LAI risperidone is an aqueous suspension of microspheres formed by a glycolic and lactic acid degradable matrix where risperidone is encapsulated [27]. More recently, a new formulation of once-monthly subcutaneous LAI risperidone has been approved by the FDA in which risperidone is suspended in ATRIGEL^®^, a polymer that solidifies in contact with tissues [28,29].

### 2.1. Pharmacokinetics

Considering the physicochemical properties of polymers of risperidone, a reduced release occurs a few days after injection (around 1% of the dose administered) followed by a lag time of 3 weeks and then a significant release is observed in weeks 4–6 [27,30]. Due to this lag phase, oral supplementation should be given in the first three weeks of treatment. Metabolism of risperidone goes through hydroxylation and N-dealkylation processes in which CY2D6 plays a predominant role in the formation of 9-OH-risperidone (9-OH-R), main active and “equipotent” metabolite [31]. Product monograph states that the half-life (t_1/2_) of risperidone plus 9-OH-R is 3–6 days [32] and the steady-state is reached after the fourth injection and maintained for 4–5 weeks after the last injection [30]. As well as t_1/2_, steady-states were determined by inspection of the concentration–time curve and not by the rule of the five t_1/2_. As Lee et al. [33] point out, this can cause an underestimation of the time when the steady state is reached and can lead to an increase in dosage and an accumulation of the drug.

More recently, a new formulation of once-monthly subcutaneous LAI risperidone has been approved by the FDA, in which risperidone is suspended in ATRIGEL^®^, a polymer that solidifies in contact with tissues, providing a sustained release. Risperidone ATRIGEL^®^ does not need oral supplementation due to its characteristic pharmacokinetic profile. After subcutaneous injection, a first peak appears 4–6 h post-dose followed by a second one 10–14 days after. Steady state is reached by the end of the second injection and the apparent half-life ranges between 8 and 9 days [28,29].

With regard to the therapeutic range of reference, the German working group for neuropsychopharmacology and pharmacopsychiatry (AGNP) Consensus Guidelines for Therapeutic Drug Monitoring established between 20 and 60 ng/mL for risperidone plus 9-OH-R [34]. This range is derived exclusively from orally given risperidone studies, extrapolating this range to patients treated with long-acting formulations, which has not yet been extensively studied. Two different studies used this AGNP range [35,36] but the other two studies differ and propose other ranges for LAI risperidone [37,38]. De Leon et al. [39] proposed an updated therapeutic range (20–30 ng/mL) for LAI risperidone as a consequence of the prolonged absorption of LAI formulations and the reduction in the administration frequency, which leads to less oscillations at steady-state conditions. Despite this, until new data about LAI risperidone are available, the AGNP therapeutic reference range is the most recommended [40].

### 2.2. Pharmacogenetics

#### 2.2.1. Efficacy

Polymorphisms in the *CYP2D6* gene could affect plasma concentrations of risperidone plus 9-OH-R (active moiety) although the product monograph reflects that concentration of the active moiety is the same in PM individuals as in other phenotypes [32]. Therefore, adjusting the dose to the patient’s phenotype is not necessary but many published studies differ on this point. It is suggested that *CYP2D6* metabolizer status cause differences in active moiety exposure and consequently in efficacy and tolerability, but there are still controversies (Table 1).

A recent meta-analysis of 15 studies involving 2125 patients taking oral risperidone concluded that *CYP2D6* activity is associated with increased exposure of both risperidone and active moiety. Risperidone steady-state plasma concentration was 2.35-fold higher in IM and 6.20-fold higher in PM, while active moiety concentration was 1.18-fold higher in IM and 1.44-fold higher in PM [52].

Furthermore, some studies suggest that PM are associated with a greater number of adverse events and treatment discontinuation which decreases clinical efficacy [53,54]. In addition, some popPK models based on oral regimens of risperidone showed large differences in CL/F depending on *CYP2D6* phenotype. Mean CL/F values reported by Feng et al. were 65.4, 36 and 12.5 L/h for EM, IM and PM, respectively [55]. The popPK model developed by Sherwin et al. [56] obtained mean values of CL/F for EM, IM and PM patients of 37.4, 29.2 and 9.38 L/h, respectively. Similar differences in CL/F between PM (three-fold lower) and EM individuals were reported by Thyssen et al. [57] in a study including pediatric and adult patients. More recently, a popPK model based on oral regimen of risperidone showed that a reduced activity of *CYP2D6* (PM) could increase up to a 106% steady-state plasma concentration and up to a 53% higher Cmax compared with NM, so dose adjustment may be necessary [58].

Nevertheless, LAI antipsychotics exhibit large differences compared to oral formulations in terms of their PK profile due to their slow and sustained absorption. To our knowledge, only a popPK LAI model of the risperidone ATRIGEL^®^ formulation has been published, which found body mass index and dose as covariates of absorption rate constants and risperidone and 9-OH-R distribution volumes, respectively. In contrast, *CYP2D6* polymorphisms were not statistically significant to any structural popPK parameter [59], which might be explained by the unbalanced and reduced number of patients recruited.

It is possible to obtain approximated information about *CYP2D6* activity using quantitative methods by the following ratios. On the one hand, the R/9-OH-R ratio (relates risperidone and 9-OH-R plasma concentrations) values <1 are normal plasma ratios that indicate normal *CYP2D6* activity (UM, NM, IM) while a value >1 indicates a lack of activity (PM) or the presence of a strong inhibitor [39,60,61]. On the other hand, total risperidone C/D ratio is a measure of drug clearance that relates the concentration of the active moiety with the dose of risperidone. The normal value for oral risperidone and for LAI formulation is approximately around 7 ng/mL per mg/day and patients with ratios >14 ng/mL per mg/day are expected to be *CYP2D6* PM [40,60]. Previous procedures could be an alternative for psychiatrists and could be a possibility for clinicians who do not have access to genotype *CYP2D6*, but it is important to take into account that these methods would only be useful when *CYP2D6* constitutes the main pathway of metabolization. It is important to take into account that the *CYP2D6* allele combination in a patient is an invariable condition; once analyzed it will not change. Thus, analyzing polymorphisms in *CYP2D6* by pharmacogenetics can be useful to select the most appropriate drugs for a patient, avoiding adverse effects or lack of efficacy concerning its *CYP2D6* metabolizer status. As a result, individualized treatment selection would become easier for prescribers.

Therefore, it seems clear that genetic variations in *CYP2D6* have an impact on plasma concentrations of risperidone, but there are controversies when it comes to relating it to the efficacy of the treatment and clinical outcome. A study of 136 patients diagnosed with schizophrenia and evaluating clinical improvement using the Positive and Negative Syndrome Scale (PANSS) did not showed association between *CYP2D6* activity variations and clinical outcome [61]. Along the same line another study of 83 drug-naïve patients experiencing a first episode of psychosis did not match the clear relation between *CYP2D6* genetic variations and clinical improvement [47]. On the contrary, another study of 76 patients with schizophrenia evaluated changes on total PANSS and *CYP2D6* polymorphism founding correlation between PMs and better response to risperidone treatment. Only three patients were classified as PMs but the power of the study was enough to establish an association with PANSS improvement. [62]. Likewise, Jukic et al. recently identified a higher incidence of therapeutic failure (discontinuation or switch to another antipsychotic) in PMs [48]. These pharmacogenetic studies are based on oral risperidone administration and not all of them had been conducted in patients with schizophrenia.

#### 2.2.2. Safety

Antipsychotics can cause hyperprolactinemia by blocking dopaminergic receptors at the tuberoinfundibular system [63]. Risperidone was reported to have a high prevalence of hyperprolactinemia among all atypical antipsychotics [64,65]. Association between *CYP2D6* and hyperprolactinemia was described in a observational study of 47 children and adolescents with autism spectrum disorder in a long-term treatment with risperidone; the number of patients with hyperprolactinemia was 100% (2/2) for PM, 47% (8/17) for IM, 48% (12/25) for NM and 0% (0/2) for UM [66]. Sex influence in hyperprolactinemia incidence has been studied, observing a higher incidence in female than in males, a study of Schoretsanitis et al. [67] involving 111 patients (61 males and 49 females) evaluates association between *CYP2D6* activity, risperidone levels and sex finding a significant association across them. Another cause of reduced patient compliance is weight gain produced by risperidone, relation between genetic polymorphisms and this adverse drug reaction is still under study. Some studies like the one performed by Lane et al. found significant association between *CYP2D6×10* allele (PM) and weight gain but data about this relation up-to-date are limited [68].

Extrapyramidal Symptoms (EPS) are the most evaluated adverse effects with *CYP2D6* in a large number of studies. Nevertheless, it is not clear the impact of *CYP2D6* variations on EPS from risperidone. Several studies showed no significant difference between these two variables and only a few find some correlation. Adverse effects were also examined in a study of 70 healthy volunteers, several genes and respective polymorphisms were associated with adverse effects (*CYP2C9*, *NAT2*, *DRD2*, *CYP2C19*) but no relation was found between *CYP2D6* polymorphisms and EPS [69]. De Leon et al. [53] showed this association in 73 patients with moderate to severe EPS and 81 patients that discontinued risperidone due to EPS. *CYP2D6* PM phenotype was associated with the first group (OR = 3.4; CI = 1.5–8.0, *p* = 0.004) and with the second group. (OR = 6; CI = 1.4–25.4, *p* = 0.02). Another recent study evaluated 22 patients and the Drug-induced Extrapyramidal Symptoms Scale (DIEPSS) score; they observed that DIEPSS score was significantly higher in the IM group (7) than in the EM group (15) [70]. A summary of LAI-risperidone pharmacokinetic and pharmacogenetic characteristics is available in Table 2. 

## 3. Long-Acting Paliperidone

Paliperidone (9-OH-Risperidone) is the primary active metabolite of risperidone. This long-acting drug is formulated as a palmitate ester of paliperidone in an aqueous suspension of nanocrystals resulting in a sustained release profile [71,72]. This technology confers an increased solubility, absorption and bioavailability [73]. Monthly formulation (PP1M) was approved by the FDA in 2009 and trimestral formulation (PP3M) in 2015, a semi-annual formulation is currently being developed [74]. For developing PP3M, the manufacturer used a PP1M population-pharmacokinetic model and avoid phase II study [75]. The main difference between PP1M and PP3M is that this last one has an increased particle size what allows its longer sustained release [76].

### 3.1. Pharmacokinetics 

Paliperidone palmitate is slowly dissolved at the injection site after the intramuscular, deltoid or gluteal, administration and then rapidly hydrolyzed. Peak plasma concentration is achieved 13 days after injection while steady-state conditions last nearly 8 months. The apparent half-life ranges between 25 and 49 days. In contrast to risperidone, paliperidone is not extensively metabolized in the liver and nearly half of the dose is excreted unchanged in urine, so dose reduction is recommended in patients with mild renal impairment [71]. LAI Paliperidone treatment is not recommended in patients with moderate to severe renal impairment.

A popPK model of LAI PP1M statistically identified BMI, creatinine clearance, injection site (deltoid vs. gluteal), injection volume and needle length as statistical covariates, demonstrating that administration procedure and the site of injection play a key role on PP1M pharmacokinetic profile [77]. The summary of product characteristics recommends a first deltoid injection of 234 mg of paliperidone palmitate followed by a second deltoid injection of 156 mg one week later [71]. Different studies evaluated therapeutical equivalence of deltoid versus gluteal injection sites. While some authors stated that differences between deltoid and gluteal injection are not clinically relevant [78], Yin et al. reported that it can compromise maintenance treatment [79]. A case report observed that absorption rate after gluteal injection decreased in obese patients due to subcutaneous fat [80]. According to this, another study estimates that time to reach steady-state differs by 4 weeks between deltoid (38w) and gluteal (42w) injection [81]. It seems that deltoid injection provides better absorption so it should be chosen instead of the gluteal zone whenever is possible, especially in obese patients.

On the other hand, PP3M is approved for use in patients previously treated with PP1M formulation for at least 4 months with a dose 3.5 times higher than the previous PP1M dosage. PP3M has a larger particle size compared to PP1M, which allows for longer sustained release and administration every 3 months. Peak plasma concentrations are achieved 30–33 days after administration while consecution of steady-state lasts nearly 15 months. Apparent half-life ranges from 84 to 95 days (deltoid administration) versus 118 to 139 days (gluteal administration). This difference in half-life of PP3M depending on site of injection could be due to flip-flop kinetics that occur on these long-acting formulations. According to Schoretsanitis et al., urgent real-world half-life studies are needed in steady-state conditions to clarify dose-dependent half-lives on dose and influence of injection site [40]. The most relevant parameters of the popPK models of PP1M and PP3M are summarized in Table 3.

With regard to the reference range, AGNP consensus guidelines established the paliperidone palmitate reference range between 20 and 60 ng/mL, but this range is again an extrapolation from oral paliperidone studies and discordance exists. A study of non-steady-state patients revealed that 45% of them were under the AGNP range [83]; on the other hand, two different studies agreed with the AGNP therapeutic range [84,85]. 

### 3.2. Pharmacogenetics 

Metabolism and elimination information provided by the manufacturer are based on a study of five healthy male subjects given a 1 mg single dose of ^14^C-paliperidone oral solution; 59% of the dose was excreted unchanged in urine. They identified four metabolic pathways but none of them metabolized more than 6.5% of the dose. Three subjects were classified as EM and two were classified as PM for *CYP2D6* using the dextrometorphan metabolic ratio. No differences in the overall plasma pharmacokinetics were observed between EM and PM. A total of 80% of the administered radioactivity was recovered in urine and 11% in the feces [86]. The small sample size (n = 5) of the study and the fact of oral administration instead of injectable does not make it possible to draw definitive conclusions with a sufficient level of evidence. Another study including 31 patients treated with paliperidone (Oral: 9 LAI: 22) did not find a statistical difference in either for the C/D ratios between different *CYP2D6* phenotypes (4 PM, 3 IM, 22 EM, 2 UM) [87]. A summary of LAI-paliperidone pharmacokinetic and pharmacogenetic characteristics is available in Table 4.

With these data it is expected that co-treatment with cytochrome inducers does not play any role in metabolism of paliperidone; however, a study performed by Yasui-Furukori et al. [88] in steady-state conditions observed that paliperidone concentrations decrease nearly 48% when carbamazepine, a potent *CYP3A4* inductor, was administered concomitantly. Due to the lack of evidence regarding the influence of *CYP2D6* polymorphisms in paliperidone plasma concentrations (Table 5), it is not expected that it could be associated with a higher risk of adverse effects.

## 4. Long-Acting Aripiprazole

Aripiprazole was the first antipsychotic to have partial agonist effects at D2 receptors. This property, plus D3 and 5-HT1A partial agonism and 5-HT2A receptor antagonism translates, into a reduction in negative, positive and cognitive symptoms of schizophrenia, minimizing the risk of some adverse effects compared with other atypical antipsychotics.

### 4.1. Pharmacokinetics

The aripiprazole once monthly (AOM) long-acting injectable is the monohydrate polymorphic form of aripiprazole [90]; currently, doses of 400 mg and 300 mg are approved for induction (along with 14 days of oral supplementation) and maintenance therapy of schizophrenia. Recently, an induction start of two injections of 400 mg has been approved, avoiding the need for oral supplementation for 14 days [90]. Due to low solubility, AOM absorption into the systemic circulation is slow and prolonged after intramuscular injection. Peak plasma concentration is reached after 4 days in deltoid injection versus 5–7 days in gluteal injection, in steady-state conditions. The mean apparent half-life is 29.9 days for 300 mg and 46.5 days for 400 mg; steady-state concentrations are achieved by the fourth injection for both sites of injection according to the manufacturer [90,91].

Systemic metabolism is carried out mainly by liver biotransformation, mediated by *CYP3A4* and *CYP2D6*, yielding to its main active metabolite dehydro-aripiprazole [92]. Dehydro-aripiprazole is also a ligand at the D2 receptor and has similar pharmacological properties to the original compound [93]. At steady state, this metabolite represents up to 40% of plasma drug concentration [94]. Therefore, both compounds are thought to contribute to the antipsychotic effects. As well as risperidone and paliperidone, aripiprazole and dehydro-aripiprazole are substrates of P-gp [95]. In this case, to our knowledge no literature is available about binding affinity to P-gp of aripiprazole and dehydro-aripiprazole. 

Recently, the new lauroxil LAI aripiprazole formulation has been approved. After intramuscular injection, aripiprazole lauroxil slowly dissolves and it is then hydrolyzed to aripiprazole. Peak plasma concentrations are achieved 41 days post-dose, while the steady-state is reached after 4 months. Supplementation with oral aripiprazole is needed 21 days after first administration. A 1-day regimen composed of 30 mg oral aripiprazole plus an intramuscular 675 mg nanocrystalline aripiprazole has also been approved. Aripiprazole lauroxil can be after administrated every 4, 6 or 8 weeks depending on the dose.

Therapeutic reference range based on oral formulation studies were reported between 100 and 350 ng/mL for aripiprazole and 150 and 500 ng/mL for the active moiety (aripiprazole plus dehydro-aripiprazole) [34]. AOM 400 mg provided sustained mean plasma concentrations comparable to those achieved by oral aripiprazole, 10–30 mg/day at steady-state. These data were evaluated in a 24-week, open-label, phase I study conducted in 41 patients with schizophrenia, receiving oral supplementation with 10 mg of aripiprazole for the first 14 days following the initial injection [91,96]. Pharmacokinetic data about AOM are to date limited; no third-party studies with PK analysis are available. 

A study performed by the manufacturer comparing deltoid and gluteal administration verified that exposure was similar between the two injection sites but absorption rate and C_max_ were higher in the deltoid group; it seems that, as in other LAI antipsychotics, deltoid administration is a better injection site [96].

Most relevant parameters of the popPK models of aripiprazole lauroxil are summarized in Table 6, which simultaneously modeled aripriprazole and dehydro-aripriprazole observations. Surprisingly, factors related with its administration (needle length or injection volume) were not statistically significant covariates in LAI-aripiprazole lauroxil pharmacokinetics, in contrast to PP1M and PP3M findings. CYP2D6 and total weight significantly affected LAI-aripiprazole pharmacokinetics.

### 4.2. Pharmacogenetics

The FDA recommends dose adjustment for aripiprazole in patients who are known *CYP2D6* PMs and AOM product monograph recommends an adjusted dose of 300 mg for this group of patients [90]. According to these data, C/D ratios observed in 62-patient study treated with oral aripiprazole indicated that PMs typically need 30–40% lower doses to achieve similar serum concentrations as NMs [98]. As aripiprazole and dehydro-aripiprazole are regarded as equipotent [93], Suzuki et al. [99] in a study of 89 healthy patients demonstrated that subjects with any or reduced functional alleles (*×5* and *×10*) for *CYP2D6* had higher C/D ratios of the active moiety of the two compounds than those without the alleles. In a recent retrospective cohort study including pharmacokinetic data of 890 patients it was found that aripiprazole active moiety exposure increased 1.6 times and 1.4 times for PMs and IMs, respectively [48]. All these data and those of other studies are available in Table 7, showing the necessary dose adjustment in PMs.

Current FDA recommendation only takes into account dose adjustment in *CYP2D6* PMs, but available studies show that dose reduction should also be carried out in *CYP2D6* IMs. In this sense, Tveito et al. [102] established that IM phenotype increases aripiprazole plasma concentration by 50% and active moiety concentration by 40%, both compared to NM. Further pharmacogenetic studies must be carried out in order to confirm this effect on plasma exposure, which could entail updating the product information regarding this group of patients. Among all the studies, only a few of them were performed with AOM, in which substantial influence of *CYP2D6* phenotype on serum concentrations was observed too, as in oral formulations [102].

Aripiprazole is generally well tolerated due to its good safety profile concerning extrapyramidal reactions which are less common than in the other atypical antipsychotics, except for akathisia [106,107]. Hendset et al. [98] observed that *CYP2D6*-defective alleles patients were associated with more potent adverse effects. In addition, a case–control study with eight patients suggested that IM and PM *CYP2D6* status are more frequently associated with extrapyramidal reactions [108]. Another study found that PM and IM were associated with higher incidence of nausea/vomiting due to higher plasma concentrations of aripiprazole in these subjects [101]. A summary of LAI-aripiprazole pharmacokinetic and pharmacogenetic characteristics is available in Table 8.

Only a few of the summarized studies are developed using LAI formulations of antipsychotics; there is an urgent need for independent and real-world LAI antipsychotics TDM studies to clarify pharmacokinetic data such as the half-life and therapeutic reference range.

## 5. Pharmacokinetics/Pharmacogenetics Implementation

Pharmacogenetics can also contribute to understand antipsychotics behavior in the organism, and this information can be totally complemented with pharmacokinetics data of LAI. The use of pharmacogenetics on treatment optimization is exemplified in different case reports [109,110,111]. Moreover, nowadays there are multiple initiatives that are already translating pharmacogenetics into clinical care around the world. To cite several examples, there is the Electronic Medical Records and Genomics (eMERGE-PGx) and St. Jude Children’s Research Hospital that are using pharmacogenetic analysis to guide treatment in patients; both initiatives are from the United States [112,113]. In Europe, the Ubiquitous Pharmacogenomics project (U-PGx) can be found, with seven countries taking part, one of them Spain [114]. In our country, there are already hospitals incorporating pharmacogenetics into health services [115]. There is also a Pharmacogenomic Society, the Spanish Society of Pharmacogenetics and Pharmacogenomics (SEFF), which is actively working on promoting pharmacogenetics knowledge and developing guidelines for healthcare professionals [116]. 

Viability of pharmacogenetics testing in psychiatry has been observed. Five years ago, two different studies were performed where physicians were asked about pharmacogenetics in psychiatry practice. Around 95% of the physicians believe that this can help them and their patients in decision-making [117] and 80–85% think that pharmacogenetics will become a standard practice in the future [117,118], which is currently a reality as aforementioned [119]. Curiously, in 2012 Mas et al. [120] studied *CYP2D6* polymorphisms in 151 patients treated with risperidone and realized that PMs received the lowest doses and UM the higher doses, without physicians knowing patients’ metabolic status. However, a preventive pharmacogenetics test would resolve the casuistry with better cost-effectiveness for the health care system and the patient versus traditional prescribing methods [121].

As we have mentioned, schizophrenia implies a great economic cost for health care systems, families and society. A study performed in patients with psychiatric conditions taking drugs not recommended based on genetic information showed that they have 69% more total health care visits, 67% more medical visits and three times more medical absence days compared to patients taking drugs recommended based on their genotypes [122]. Another study associated *CYP2D6* PMs and UMs to longer hospital stay duration compared to EMs [123]. In 2013, Herbild et al. [124] demonstrated cost-effectiveness of preemptive *CYP2D6* or *CYP2C19* genotyping in patients with schizophrenia. TDM and preemptive genotyping of patients could reduce the time spent seeking the right antipsychotic drug and dose, minimizing hospital stay and visits, preventing adverse drug reactions and reducing associated costs. To all of these expenses must be added the expensive treatment with LAI, so studies focused on therapies with this group of drugs are needed to assess the cost–benefit ratio of preemptive *CYP2D6* genotyping.

## 6. Unsolved Questions and Future Directions 

According to the LAI risperidone product monograph, 9-OH-R has similar pharmacological activity to risperidone [32]; however, oral paliperidone has been approved with twice the daily dose of risperidone. Accordingly, de Leon et al. [60,125] suggest that risperidone is twice as potent as paliperidone. In this sense, brain imaging studies showed that lower doses of risperidone cause a similar percentage of binding to higher doses of paliperidone [126,127]. This could be explained by the high affinity of paliperidone to P-gp, minimizing blood–brain barrier (BBB) penetration.

The correlations between plasma and brain concentration for risperidone and 9-OH-R have been studied by Aravagiri et al. [128] in rats documenting differences in the distribution of both compounds in the brain, showing plasma/brain ratios of 6.7 for risperidone and 32.9 for 9-OH-R. These differences could be explained by two different factors: first, affinity of risperidone and 9-OH-R for P-glycoprotein (P-gp) in BBB. Wang et al. [129] conducted a study in abcb1a/b knockout mouse model dysfunctional in P-gp. Data showed that the ratio of plasma to brain of 9-OH-R was 2.4-fold higher than that for risperidone. This could mean that 9-OH-R presents a higher affinity for P-gp than risperidone and it could explain the greater toxicity of risperidone than 9-0H-R [60]; second, hydroxylation of risperidone to 9-OH-R makes it more hydrophilic, decreasing the ability to cross the BBB.

A preliminary assessment has demonstrated the prediction performance of D2/3 receptor occupancy a priori to optimize the dosing regimen based on risperidone blood concentrations. Accordingly, a 65–80% D2/3 receptor occupancy is associated with maximal effectiveness, minimal risk of adverse events and reduced risk of relapse in patients receiving supra-therapeutic doses [130]. Similar D2/3 receptor occupancy was also reported by Shin et al. after aripiprazole administration, demonstrating the improvement of the cognitive function in a small sample size of patients with schizophrenia [131]. Therefore, predictive popPK models are encouraged to better characterize the PK properties of LAI antipsychotics and identify the sources of inter-individual variability in order to establish a PK/PD framework able to assess D2/3 receptor occupancy. The mathematical framework would allow the evaluation of dosing strategies in special sub-groups of patients, which will help to achieve an optimal efficacy/safety balance.

## 7. Conclusions

Atypical LAI have resulted in a great increase in safety compared with the previously available typical LAI. However, limited evidence has restricted the proper characterization of their pharmacokinetic properties, specifically regarding LAI of risperidone, paliperidone and aripiprazole. The role of CYP2D6 has been demonstrated on the PK of LAI aripriprazole, although it remains uncertain for LAI risperidone and LAI paliperidone. In spite of that fact, the developed popPK models showed common aspects regarding the influence of body weight, administration site and needle characteristics on the pharmacokinetic behavior of these drugs. However, the reduced number of patients enrolled, the prolonged drug half-life and the limited number of clinical studies available may limit the identification of covariates and pharmacogenetic effects that would help to explain the large inter-individual differences concerning drug exposure. Pharmacogenetic analysis should be considered with caution because the CYP2D6 polymorphisms analyzed in each study are different. Assuming that all the polymorphisms that were not analyzed are wild-type allele; consequently, exceedance of NM would be obtained. The CYP2D6 gene is a complex gene, which presents duplications, deletions and even non-functional hybrids and, therefore, requires a more exhaustive approach to be able to determine the relationship between kinetics and genetics. Additional evaluations, including continuous PK endpoints, are encouraged to provide a more comprehensive characterization of the PK/PD properties of LAI in order to guide the individual dose selection.

## Figures and Tables

**Table 1:** Studies between *CYP2D6* phenotype and relation with active moiety exposure.

Study	LAI/Oral	n	Race	Age (Median)	Male/Female	*CYP2D6* Phenotypes	Outcome
Vermeulen A et al. [41]	Oral	407	NR	38	267/140	PM/IM/NM	Irrelevant
Scordo MG et al. [42]	Oral	37	Caucasians	41	30/7	PM/IM/NM/UM	Irrelevant
*Cho* HY et al. [43]	Oral	24	Asian	24.6	NR	PM/NM	Irrelevant
Hendset, M et al. [35]	LAI	90	Caucasians	38	53/37	PM/IM/NM/UM	Relevant (higher plasma exposure for IM and PM)
Llerena A et al. [44]	Oral	35	Caucasians	43	NR	PM/IM/NM/UM	Irrelevant
Choong, E et al. [45]	LAI	42	Caucasian (76%)	35	30/12	PM/IM/NM/UM	Relevant (higher plasma exposure for IM and PM and lower for UM)
Leon, J. D et al. [46]	Oral	277	Caucasian (78%)	43.7	150/127	PM/IM/NM/UM	Relevant (higher plasma exposure for PM)
Jovanović, N et al. [47]	Oral	83	Caucasians	30.3	17/66	PM/NM/IM	Irrelevant
Jukic, M et al. [48]	Oral	725	Caucasians	42.8	355/370	PM/IM/NM/UM	Relevant (higher plasma exposure for IM and PM and lower for UM)
Locatelli, I et al. [49]	LAI	50	Caucasian	30	39/11	PM/IM/NM/UM	Irrelevant
Vandenberghe, F et al. [50]	Oral	150	Caucasian (81%)	39	82/68	PM/IM/NM/UM	Relevant (higher plasma exposure for PM)
Gunes, A et al. [51]	Oral	46	Caucasian	45	35/11	PM/NM/PM	Relevant (higher plasma exposure for PM)

Classified in relevant (significative association between CYP2D6 phenotype and active moiety exposure) or irrelevant (no significative association between CYP2D6 phenotype and active moiety exposure or incorrect assignment of CYP2D6 genotype due to missing copy number variation analysis). Abbreviations: PM, Poor Metabolizer; IM, Intermediate Metabolizer; NM, Normal Metabolizer; UM, Ultra Metabolizer; NR: Not reported. Those studies that do not analyze the number of copies of the gene have been classified as irrelevant. Among those classified as relevant, it is important to highlight that they should be taken as a guideline, taking into account that the main limitation is the high heterogeneity that exists between the different methods of analysis used, as well as the polymorphisms explored in the determination of the phenotype of CYP2D6.

**Table 2:** Summary of PK/PG characteristics of LAI-Risperidone.

Drug	Main Active Metabolite	Therapeutic Reference Range	Metabolism	TDM Recommendation (33)	Mean PK Values	Genetic Test Recommendation (94)
LAI-Risperidone microespheres	9-OH-Risperidone	20–60 ng/mL	*CYP2D6*/*CYP3A4*	2	T_max_: 28 days T_ss_: 8 weeks T_1/2_: 3–6 days	Informative
LAI-Risperidone ATRIGEL^®^	9-OH-Risperidone	20–60 ng/mL	*CYP2D6*/*CYP3A4*	2	Double T_max_: 4–6 h and 10–14 days T_ss_: 8 weeks T_1/2_: 8–9 days	Informative

Abbreviations: T_max_, time to maximum concentration after administration; T_ss_, time to reach steady state; t_1/2_, apparent half-life of elimination. TDM Levels of recommendation: 1: Strongly recommended; 2: Recommended; 3: Useful; 4: Potentially useful (33). Genetic test; informative: the label contains information stating that a particular gene affects or does not effect drug efficacy, not clinically significant.

**Table 3:** Population pharmacokinetic models of paliperidone palmitate.

Author	Formulation	Model	Covariates	Parameters Values	Equations
Samtani et al. [77]	LAI-PP1M	- One-compartment model with first-order elimination - Dual and sequential input absorption: rapid zero order followed by first-order absorption after a lag time. - Flip-flop kinetics due to dissolution rate limited absorption.	- SEX, AGE, IVOL and INJS on Ka - CLCR on CL - BMI and SEX on Vd	KA: 0.488 h^−1^ CL: 4.95 L/h VD: 391 L	$\mathrm{CL}=4.95\times {\left(\frac{\mathrm{CLCR}}{110.6}\right)}^{0.376}$ $V=\mathrm{SEX}\times 391\times {\left(\frac{\mathrm{BMI}}{26.8}\right)}^{0.889}$ SEX = 1 for males SEX = 0.726 for females
Magnusson et al. [82]	LAI-PP3M	- One-compartment model with first-order elimination - Dual and sequential input absorption processes: rapid zero absorption followed by first-order absorption.	- IVOL on Ka - INJS and SEX on Ka_max_ - CLCR on CL - BMI on Vd	KA: not available. CL/F: 3.84 L/h VD/F: 1960 L	$\mathrm{CL}=3.84\times {\left(\frac{\mathrm{CLCR}}{115}\right)}^{0.316}$ $V=1960\times {\left(\frac{\mathrm{BMI}}{26.15}\right)}^{1.18}$

Abbreviations: LAI: long-acting injectable. PP1M: once-monthly paliperidone palmitate. PP3M: once every 3 months paliperidone palmitate. IVOL: injection volume. INJS: injection site. BMI: body mass index. NDLL: needle length. KA: first-order absorption rate constant. Ka_max:_ maximum absorption rate. CL: clearance. VD: apparent volume of distribution. CL/F: clearance relative to bioavailability (in the absence of intravenous administration). VD/F: apparent volume of distribution relative to bioavailability (in the absence of intravenous administration).

**Table 4:** Summary of PK/PG characteristics of LAI-Paliperidone.

Drug	Main Active Metabolite	Therapeutic Reference Range	Metabolism	TDM Recommendation (33)	Mean PK Values	Genetic Test Recommendation (94)
LAI-Paliperidone PP1M	-	20–60 ng/mL	60% excreted unmetabolized/ *CYP2D6*/*CYP3A4* In vitro	2	T_max_: 13 days T_ss_: 8–9 months T_1/2_: 25–49 days	Informative
LAI-Paliperidone PP3M	-	2–60 ng/mL	60% excreted unmetabolized/ *CYP2D6*/*CYP3A4* In vitro	2	T_max_: 30–33 days T_ss_: 15 months T_1/2_: 84–95 days (deltoid) 118–139 days (gluteal)	Informative

Abbreviations: T_max_, time to maximum concentration after administration; T_ss_, time to reach steady state; t_1/2_, apparent half-life of elimination. TDM Levels of recommendation: 1: Strongly recommended; 2: Recommended; 3: Useful; 4: Potentially useful (33). Genetic test; informative: label contains information stating that a particular gene affects or does not affect drug efficacy, not clinically significant.

**Table 5:** Studies between *CYP2D6* phenotype and the relation with paliperidone plasma exposure.

STUDY	Oral/LAI	n	Race	Age (Median)	Male/Female	*CYP2D6* Phenotypes	Outcome
Vermeir et al. [86]	Oral	5	Caucasian	51	5/0	PM/NM *	Irrelevant
Lisbeth et al. [87]	Oral/LAI	31	Caucasian	35	22/9	PM/IM/EM/UM	Irrelevant
Berwaerts et al. [89]	Oral	60	Caucasian (75%)	NR	60/0	NM/UM	Irrelevant (Coadministration with paroxetine)

Irrelevant outcome (no significative association between CYP2D6 phenotype and paliperidone plasma exposure or incorrect assignment of CYP2D6 genotype due to missing copy number variation analysis). Abbreviations: PM, Poor Metabolizer; IM, Intermediate Metabolizer; NM, Normal Metabolizer; UM, Ultra Metabolizer; NR: Not reported. * Phenotyped using the dextromethorphan metabolic ratio.

**Table 6:** Population pharmacokinetic model of LAI aripiprazole.

Author	Formulation	Model	Covariates	Parameters Values	Equations
Hard et al. [97]	Aripiprazole lauroxil	- 2-compartment model with sequential zero-order absorption followed by a first-order process - Zero-order conversion of aripiprazole lauroxil to aripiprazole	- CYP2D6 PM on CL - Total weight on Vd	KA: 0.574 h^−1^ CL/F: 0.767 L/H (PM) vs. 2.02 L/h (non-PM) VD/F: 2122 L	CL equation not reported $\mathrm{VD}=268\times \left(\frac{\mathrm{WT}}{70}\right)$

Abbreviations: KA: first-order absorption rate constant. CL/F: clearance relative to bioavailability (in the absence of intravenous administration). VD/F: apparent volume of distribution relative to bioavailability (in the absence of intravenous administration).

**Table 7:** Studies between *CYP2D6* phenotype and the relation with aripiprazole plasma exposure.

Study	Oral/LAI	n	Race	Age (Median)	Male/Female	*CYP2D6* Phenotypes	Outcome
Suzuki et al. [99]	Oral	89	Asian	38	46/43	IM/NM/UM	Relevant (higher plasma exposure for IM and PM)
Suzuki et al. [100]	Oral	63	Asian	NR	36/33	PM/IM	Relevant (For ×10 allele)
Hendset et al. [54]	Oral	266	Caucasian	33	NR	IM/NM	Irrelevant
Hendset et al. [98]	Oral	62	Caucasian	31	29/33	PM/NM	Relevant (higher plasma exposure for PM)
Belmonte et al.* [101]	Oral	148	Caucasian	26	85/63	PM/IM/NM/UM	Relevant (higher plasma exposure for PM and IM)
Tveito et al. [102]	Oral/LAI	635(469/166)	Caucasian	40	294/341	PM/IM/NM/UM	Relevant (higher plasma exposure for PM and IM)
Jukic et al. [48]	Oral	890	Caucasian	37	400/490	PM/IM/NM/UM	Relevant (higher plasma exposure for PM and IM)
Lisbeth et al. [87]	Oral/Lai	18(17/1)	Caucasian	36	11/7	PM/IM/NM/UM	Relevant (higher plasma exposure for PM)
van der Weide et al. [103]	Oral	130	Caucasian	NR	NR	PM/IM/NM/UM	Irrelevant
Azuma et al.** [104]	Oral	27	Asian	NR	NR	IM/NM	Relevant (Coadministration with CYP2D6 inhibitors)
Kubo et al.** [105]	Oral	20	Asian	24	20/0	IM/NM	Irrelevant

Classified in relevant (significative association between CYP2D6 phenotype and active moiety exposure) or irrelevant (no significative association between CYP2D6 phenotype and active moiety exposure or incorrect assignment of CYP2D6 genotype due to missing copy number variation analysis). Abbreviations: PM, Poor Metabolizer; IM, Intermediate Metabolizer; NM, Normal Metabolizer; UM, Ultra Metabolizer; NR: Not reported. * Those studies which do not analyze the number of copies of the gene have been classified as irrelevant. Among those classified as relevant, it is important to highlight that it should be taken as a guideline, taking into account that the main limitation is the high heterogeneity that exists between the different methods of analysis used, as well as the polymorphisms explored in the determination of the phenotype of CYP2D6. ** Single-dose study.

**Table 8:** Summary of PK/PG characteristics of LAI-Aripiprazole.

Drug	Main Active Metabolite	Therapeutic Reference Range	Metabolism	TDM Recommendation (33)	Mean PK Values	Genetic Test Recommendation (94)
LAI-Aripiprazole Monohydrate	Dehydro-aripiprazole	100–350 ng/Ml Active moiety: 150–500 ng/mL	*CYP2D6*/*CYP3A4*	2	T_max_: 4–7 days T_ss_: 4 months T_1/2_ 400 mg: 46.5 days 300 mg: 29.9 days	Actionable
LAI-Aripiprazole Lauroxil	Dehydro-aripiprazole	100–350 ng/mL Active moiety: 150–500 ng/mL	*CYP2D6*/*CYP3A4*	2	T_max_: 41 days T_ss_: 4 months T_1/2_: 53.9–57.2 days	Actionable

Abbreviations: T_max_, time to maximum concentration after administration; T_ss_, time to reach steady state; t_1/2_, apparent half-life of elimination. TDM Levels of recommendation: 1: Strongly recommended; 2: Recommended; 3: Useful; 4: Potentially useful (33). Genetic test; Informative: this label contains information stating that a particular gene affects or does not affect drug efficacy, not clinically significant; Actionable: this label may contain information about changes in efficacy due to gene variants.

## Data Availability

Not applicable.
